# “Take the Rough With the Smooth”: Modesty Modulates Neurocognitive and Emotional Processing of Social Feedback

**DOI:** 10.1002/hbm.70395

**Published:** 2025-10-29

**Authors:** Xin Wang, Chuhua Zheng, Yanhong Wu

**Affiliations:** ^1^ School of Psychological and Cognitive Sciences Peking University Beijing China; ^2^ Institute of Scientific and Technical Information of China Beijing China; ^3^ Beijing Key Laboratory of Behavior and Mental Health Peking University Beijing China; ^4^ State Key Laboratory of General Artificial Intelligence Peking University Beijing China

**Keywords:** emotion regulation, fMRI, modesty, social feedback, social judgment paradigm

## Abstract

Self‐enhancement motivates individuals to prefer positive or expected social feedback over negative or unexpected feedback, thereby eliciting corresponding emotional experiences. Emotion regulation strategies that aim to reduce negative experiences and enhance positive ones often face the dilemma of prioritizing one outcome at the expense of the other. Modest individuals, characterized by the low self‐focus perspective, may demonstrate advantages in managing emotional experiences derived from self‐relevant social feedback. In this study, participants with high and low levels of modesty were scanned with functional magnetic resonance imaging while receiving social feedback of different valences and congruencies, with feedback indicating whether others liked participants. Results showed that highly modest individuals were less likely to use expressive suppression as an emotion regulation strategy. At the neural level, trait modesty modulated brain activity in the inferior parietal lobe and left superior temporal gyrus under unexpected conditions compared to expected conditions, as well as in the ventral anterior cingulate cortex, ventral medial prefrontal cortex, dorsal anterior cingulate cortex, and dorsolateral prefrontal cortex under acceptance versus rejection conditions. Psychophysiological interaction analysis and brain‐behavior correlation analyses further explored the mechanisms of modesty, helping individuals reduce negative experiences and enhance positive experiences. Our findings reveal the cognitive processing patterns and brain activity of modest individuals when dealing with social feedback and provide insights into how individuals can better cope with social feedback.

## Graphical abstract

While social feedback is crucial to individuals’ self‐concept, individuals persistently display positive bias and experience emotion regulation difficulties. Results revealed that modest individuals demonstrate dual competence in both reducing negative experiences and enhancing positive experiences during self‐relevant information processing.

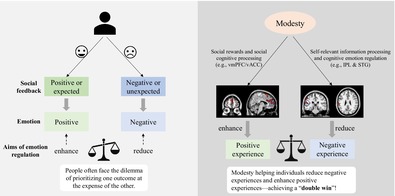

## 1 Introduction

An important question in emotion regulation is whether individuals can achieve a “double win”: experiencing more positive emotions and fewer negative ones (Gross 2015). However, this is difficult to achieve when processing self‐relevant information due to self‐enhancement bias, which leads people to perceive themselves positively (Alicke and Sedikides 2009; Dufner et al. 2019). In social interactions, a typical example of self‐relevant information is social feedback, which refers to different evaluative information about themselves or their behavior provided by others (Chen et al. 2024; Lundgren 2004). Clearly, people tend to prefer positive or expected social feedback, and exhibit positive experiences after receiving such information (Poore et al. 2012; Somerville et al. 2006). Conversely, negative or unexpected feedback often causes negative experiences (Miyamoto et al. 2014) and contributes to psychological distress or mental health issues (Guo et al. 2022; Zhang et al. 2023). To achieve better emotional experiences, individuals often employ various regulation strategies (Gross 2002, 2015). However, these strategies either simultaneously reduce both positive and negative experiences (e.g., suppression; Fernandes and Tone 2021; Miyamoto et al. 2014), or amplify both positive and negative experiences (e.g., social sharing; Brans et al. 2013), and may even decrease positive experiences while increasing negative ones (e.g., rumination; Brans et al. 2013). Thus, when processing self‐relevant information, is it possible for individuals to achieve the “double win”?

Returning to the origin of the problem, this dilemma arises from individuals’ self‐enhancement. There exists a group of people who are generally considered less prone to self‐enhancement—modest individuals. Previous research has primarily conceptualized modesty as a personality trait, with its core lying in the ability to adopt a low self‐focus perspective while recognizing and valuing the presence and contributions of others (Nadelhoffer and Wright 2017; Peterson and Seligman 2004). Low self‐focus indicates that modest individuals examine themselves within the context of a larger world, pay less attention to themselves, and recognize their own limitations (Nadelhoffer and Wright 2017; Wang et al. 2025). Consequently, modest individuals are able to overcome self‐enhancement bias, possess clearer self‐awareness, demonstrate higher openness to experiences, and exhibit greater adaptability in social interactions (Davis et al. 2010; Nadelhoffer and Wright 2017; Owens et al. 2013; Peterson and Seligman 2004; Tangney 2000).

Based on the characteristics of modest individuals, particularly the low self‐focus, we propose that they experience fewer negative emotions and remain calmer when facing negative or unexpected social feedback. Generally, due to self‐enhancement, people pay more attention to self‐related information and experience more intense negative emotions and surprise when facing such negative information. This has also been confirmed by previous neuroimaging studies. Research shows negative feedback significantly activates brain regions associated with social pain processing, such as the superior parietal lobe (SPL) and the cingulate cortex (Eisenberger et al. 2003; Zeidan et al. 2015). Unexpected feedback tends to cause stronger activation in brain regions related to expectation and cognitive monitoring, such as the dorsal anterior cingulate cortex (dACC) and the inferior parietal lobe (IPL) (Somerville et al. 2006; Yan et al. 2023). When faced with these negative experiences, some individuals tend to employ the expressive suppression strategy (Brans et al. 2013). However, the effects of suppression on reducing negative experiences have been reported inconsistently (effective or non‐significant), and it could even lead to a decrease in positive emotions (Brans et al. 2013; Fernandes and Tone 2021; Miyamoto et al. 2014). Compared to less modest individuals, modest individuals adopt a low self‐focus perspective (Nadelhoffer and Wright 2017; Peterson and Seligman 2004), approach critical feedback with a calm and open attitude (Davis et al. 2010; Owens et al. 2013), and are more willing to accept information that might challenge their self‐concept (Chancellor and Lyubomirsky 2013; Tangney 2000). Therefore, we hypothesize that modest individuals would experience fewer negative emotions and be less likely to adopt the suppression regulation strategy when facing negative experiences.

Modesty may also moderate individuals’ responses to positive or expected social feedback. Previous research shows that positive or expected feedback activates brain regions related to social rewards, such as the ventral anterior cingulate cortex (vACC) and the ventral medial prefrontal cortex (vmPFC) (Poore et al. 2012; Somerville et al. 2006), which aligns with the positive bias when processing self‐relevant information. Modest individuals possess more accurate self‐awareness, yet this does not imply diminished positive experiences upon receiving favorable feedback. Conversely, theoretical and empirical studies indicate that modest individuals value their social connections with others, demonstrating heightened prosocial and cooperative behaviors in social interactions (Peterson and Seligman 2004; Zheng et al. 2022). Therefore, positive experiences should also be social rewards for modest individuals, and they would at least not experience fewer positive affects when receiving positive or expected feedback.

In addition, the influence of modesty on cognitive and emotional regulation when facing social feedback may also be reflected through functional connectivity. Therefore, the study planned to employ psychophysiological interaction (PPI) analysis (Friston et al. 1997) to investigate the positive role of trait modesty in processing feedback information. As a widely accepted approach to estimate task‐modulated functional connectivity, PPI measures whether and how functional connectivity varies with experimentally manipulated psychological variables (Li et al. 2022). Previous studies have demonstrated that the vmPFC/vACC plays a key role in social rewards (Poore et al. 2012; Somerville et al. 2006), and its connectivity with other brain regions has been linked to the cognitive reappraisal of negative emotions (Doré et al. 2017). Thus, the current study would examine the functional connectivity of the vmPFC/vACC with other regions to further investigate the effects of modesty.

In sum, we propose that modesty can help individuals achieve a “double win” in emotion regulation when processing self‐relevant information. In other words, we hypothesize that modest individuals demonstrate dual competence in both reducing negative experiences and enhancing positive experiences. To verify this, the current study employed the Social Judgment Paradigm (SJP, Somerville et al. 2006) combined with fMRI to explore how modesty moderated cognitive emotional responses and brain activity in the processing of social feedback with various valences and congruencies. We hypothesize that positive feedback would lead to greater activation in brain regions associated with social rewards, while negative feedback will significantly activate brain regions involved in processing social pain. Additionally, unexpected feedback would lead to greater activation in brain regions related to expectation and cognitive monitoring. Moreover, we hypothesize that modesty would modulate these activations, reflecting stronger emotion regulation abilities. Finally, we hypothesize that PPI analyses would show associations between trait modesty and functional connectivity of the vmPFC/vACC with other regions, helping to clarify the mechanisms of modesty in social feedback processing.

## 2 Materials and Methods

### 2.1 Participants

Participants were 54 Chinese adults randomly recruited from Peking University. However, two participants were excluded due to excessive head motion (> 2 mm in any direction), and five participants were excluded due to having too few trials in a specific experimental condition. The valid sample included 47 participants (22 male, 25 female), ranging from 18 to 28 years old (M = 21.40, SD = 2.47). Only one participant was left‐handed. Participants provided informed consent and could receive a certain reward after the experiments. All procedures were approved by the Committee for Protecting Human and Animal Subjects of the School of Psychological and Cognitive Sciences, Peking University.

### 2.2 Questionnaires

The eight‐item Modesty Subscale from the Honesty‐Humility HEXACO measure (Lee and Ashton 2004) was used to measure trait modesty. This subscale conceptualizes modesty as a tendency to avoid self‐enhancement and status seeking, well reflecting the core characteristics of modesty. It has demonstrated consistent results with experimentally priming individuals' modest states (Wang et al. 2025), and has been widely used in prior research on modesty across different cultural samples (e.g., Teo et al. 2023; Wang et al. 2025). Additionally, the Rosenberg Self‐esteem Scale (Rosenberg 1965) and the Positive Affect and Negative Affect Schedule (Watson et al. 1988) were used to measure self‐esteem and emotional state. The Emotional Regulation Questionnaire (Gross and John 2003) was used to measure emotion regulation strategies. The 12‐item Brief Fear of Negative Evaluation Scale–Revised (Carleton et al. 2006) and the 19‐item Social Interaction Anxiety Scale (Mattick and Clarke 1998) were used to measure fear of negative evaluation and anxiety levels.

### 2.3 Experimental Design and Procedure

We adopted SJP (Somerville et al. 2006) combined with fMRI to explore the cognitive and emotional responses of individuals when receiving different feedback. In this paradigm, participants were led to believe that their photos were presented to a group of peers for evaluation. Specifically, they were told that these peers would view their photos and indicate whether they “liked” or “disliked” them based on first impressions. The use of the SJP induces positive/negative emotions through “like/dislike” feedback and creates expected/unexpected conditions by comparing feedback with the participants’ expectations, allowing a clear dissociation between feedback valence and expectancy. As the SJP has been widely used in the study of social feedback (e.g., Somerville et al. 2006; Van der Molen et al. 2014), adopting this binary feedback enables direct comparison with previous studies. The simple structure also provides computational efficiency and flexibility for testing moderating effects of personality traits (e.g., Van der Molen et al. 2014), thereby facilitating the examination of how modesty modulates responses to social feedback in the current study. The entire experiment comprised the following parts.

#### 2.3.1 Interviews

First, the participants were interviewed about some personal information such as hobbies and personality characteristics, which lasted about 10 min. After the interview, all participants were asked to take a portrait photograph and complete a self‐report questionnaire. They were also told that a group of peers would look at their photos and profiles and rate them comprehensively based on the first impressions they formed.

#### 2.3.2 Functional Magnetic Resonance Imaging Social Judgment Task

After about 1 to 2 weeks, the participants were invited to participate in the experiment. Each participant was told that a total of 200 evaluators had completed his/her comprehensive evaluation, and the final number of qualified effective evaluators was 160. Before the formal experiment, there were 10 trials in the practice part, and the photos used in the practice were no longer used in the formal experiment. In each trial, participants would see the photo of an evaluator and need to predict whether he/she “like” them. The evaluator's evaluation feedback result was then presented as “like” or “dislike.” In addition, participants were asked to rate their self‐esteem and emotional state before and after the experiment.

#### 2.3.3 Trial Details

**FIGURE 1:**
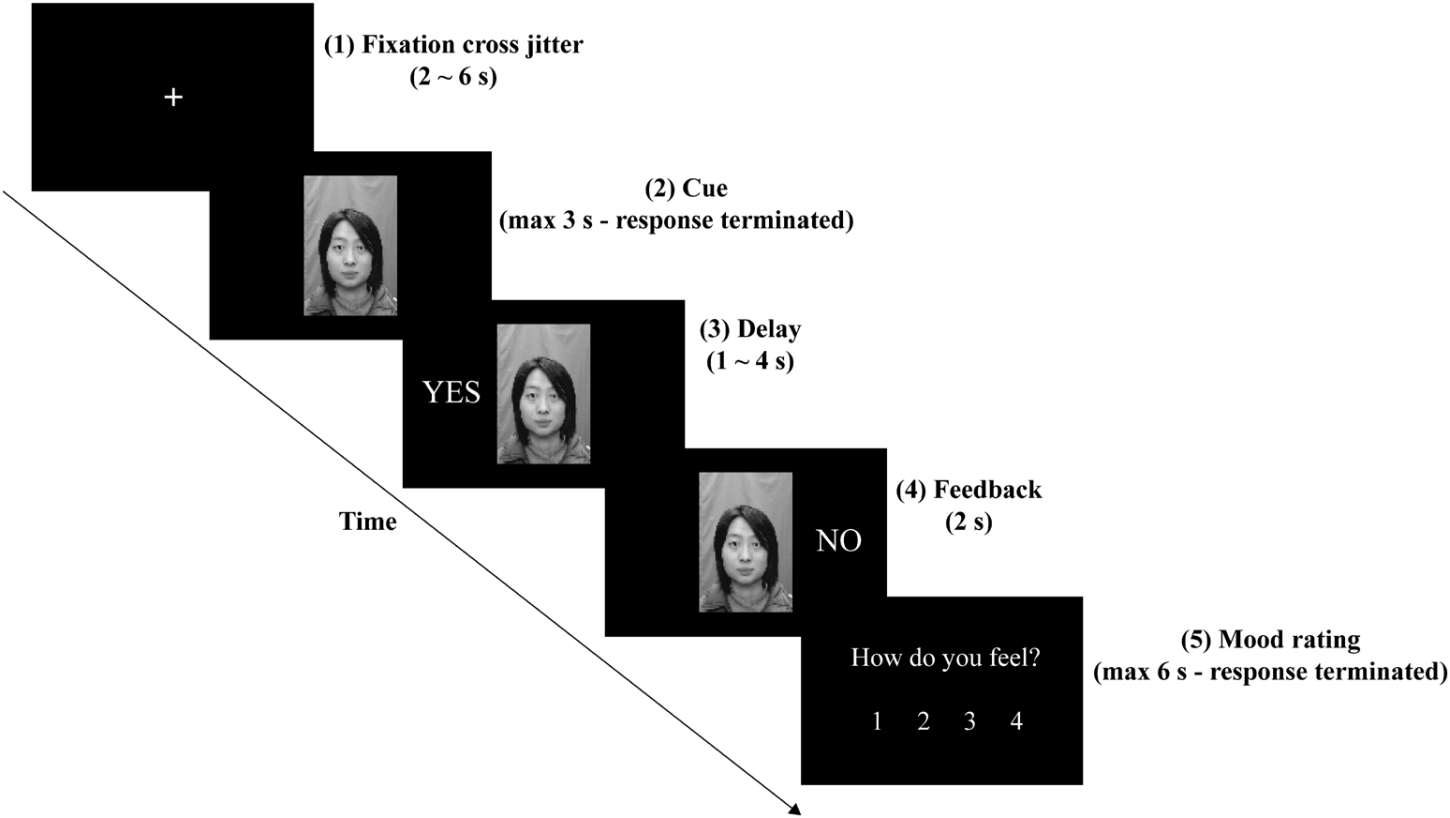
A sample trial in the Social Judgment Paradigm. Each trial comprised five stages: (1) Fixation cross jitter; (2) Cue: Participants decide whether a peer on the screen would like (YES) or dislike (NO) them; (3) Delay: The response (judgment) of the participant is shown on the left of the face; (4) Feedback: The participant receives feedback on whether the peers liked them or not. This feedback is either expected (YES‐YES/NO‐NO) or unexpected (YES‐NO/NO‐YES), and is presented on the right of the face. (5) Mood rating: The participant rates how they feel after receiving the feedback, ranging from “1” (very unhappy) to “4” (very happy).

The experiment used an event‐related design, with five runs of scan. Each run included 32 trials, and each trial comprised fixation cross jitter, cue, delay, feedback, and mood rating (see Figure 1). The participants were asked to evaluate their current mood after receiving the feedback, ranging from 1 (very unhappy) to 4 (very happy).

#### 2.3.4 After the Scanning Task

The participants were asked to rate their self‐esteem, emotional state, and how much acceptance feedback they had received (0%–100%) after finishing the scanning task. Besides, participants were asked to recall their thoughts about the process of the experiment and write down the purpose of the experiment. Finally, after all the participants had completed the experiment, the deception involved in the study and the true intention of the experiment were explained.

### 2.4 Magnetic Resonance Imaging Data Acquisition

Participants were scanned using the 3‐T Siemens Prisma system scanner with a 64‐channel standard head coil. Functional images were collected using a T2‐weighted, gradient‐echo, and echo planar image (EPI) imaging sequence. Time repetition (TR) was 2000 ms, time echo (TE) was 30 ms, and flip angle (FA) was 90°. Each whole brain image included 62 layers of cross‐sectional scanning. The interlayer matrix was 112 × 112 and the field of view (FOV) was 224 × 224 mm^2^, with a 3 × 3 × 4 mm^3^ spatial resolution. Before functional image scanning, each participant was scanned with a high‐resolution T1‐weighted structural image (TR = 2530 ms, TE = 2.98 ms, FA = 7 degrees) with a 0.5 × 0.5 × 1.0 mm^3^ spatial resolution.

### 2.5 Functional Magnetic Resonance Imaging Preprocessing

Matlab Statistical Parametric Mapping (SPM) 12 was used to preprocess functional images. Participants with head motion exceeding 2 mm in any direction were excluded. The pre‐processing includes the following steps: (1) In order to reduce the influence of magnetic field instability on the data at the beginning of scanning, images with a fixation of 30 s before and after each block were removed; (2) Layers of each slice were corrected for the difference in acquisition timing; (3) Functional images were realigned to the first slice to correct the artifacts caused by head movement, and generate six movement parameters (x, y, and z for translation; pitch, roll, and yaw for rotation); (4) Functional images of each subject were normalized to the standard space of Montreal Neurological Institute (MNI), and each voxel of the functional images was resampled into cube voxels of 2 × 2 × 2 mm^3^ size; (5) Functional images were smoothed in space by Gaussian filter with full‐width at half maximum (FWHM) of 8 mm.

### 2.6 Whole Brain: Multilevel Model

Given that we differentiated trials based on participants’ judgments, the number of observations in each cell of the study design was unequal. Referring to the analysis of Nohlen et al. (2019), we constructed a multilevel model (MLM) capable of handling such unbalanced designs, allowing us to estimate the effects of trial type and participants’ judgments simultaneously. At the individual (first) level, we specified a general linear model (GLM). The feedback events of expected acceptance, unexpected rejection, unexpected acceptance, and expected rejection were modeled as regressors. We also included the following nuisance regressors to control for non‐experimental variance: cue (displaying the peer's photo), delay (the waiting interval between prediction and feedback), and mood rating (trial‐wise rating of self‐reported mood after feedback). Head motion in all participants was less than 2 mm in any direction. Trials in which participants failed to respond in time were excluded from the analysis. At the group (second) level, a random‐effects model was used to explain the variation between participants, and the overall inference was made. First, we performed a single‐sample *t*‐test on these contrast activation maps to locate the brain regions involved in social feedback processing. Second, in order to test the moderating effect of trait modesty, we used contrast activation maps at the first level to establish multiple regression models, and included trait modesty, age, gender, and self‐esteem as covariables in the model.^1^ For our analyses, we used a stringent priori threshold of *p* < 0.001 with a cluster size of *p* < 0.05 (FWE).

### 2.7 Psychophysiological Interaction Analysis

To further investigate how trait modesty influences individuals' cognitive‐emotional processing, PPI analysis was conducted to examine the functional connectivity between the vmPFC/vACC and other brain regions. The vmPFC/vACC identified in whole‐brain analysis was used as seed points, with a radius of 6 mm sphere centered on peak coordinates. Time series were extracted using the design matrix of the GLM at the individual level, which mainly included three regression factors: (1) Signals extracted from seed brain regions (physiological variables); (2) An event‐related temporal variable (psychological variables). For example, the variable was 1 when the “acceptance” feedback appeared and was −1 when the “rejection” feedback appeared; (3) The physiological variables were multiplied with the psychological variables to obtain the psychophysiological interaction terms (PPI item). In addition, the model also included four dummy variables to balance the chunking effect as irrelevant control variables, along with six head motion parameters. A one‐sample *t*‐test was conducted for second‐order PPI group analysis, in which trait modesty was a covariate. Significant effects were reported using a combined voxel‐level threshold of *p* < 0.001 (uncorrected) and cluster‐level threshold of *p* < 0.05 (corrected for family‐wise error). One participant was excluded from the PPI analysis because the individual's region of interest could not be located in vmPFC/vACC.

### 2.8 Brain Behavior Correlation

Multiple regressions were used to explore the relationship between activated regions and changes in mood before and after the experiment. The score of emotion regulation strategies was used as the variable of interest. Whole brain cluster‐level FWE was used for multiple comparisons. The significance threshold for FWE was set at *p* < 0.05 (cluster‐forming threshold at voxel‐level: *p* < 0.001).

## 3 Results

### 3.1 Behavior Analysis

#### 3.1.1 Modesty and Emotional Regulation

The test questions after the experiment showed that all participants believed in the manipulation of social acceptance and social rejection, and had no doubt about the purpose of the experiment. As shown in Table 1, trait modesty was significantly negatively correlated with expressive suppression (*r* = −0.322, *p* = 0.027), indicating that the more modest people were, the less they used expressive suppression.

**TABLE 1:** Results of correlation analysis.

	Mean (SD)	1	2	3	4	5	6	7
1. Age	21.40 (2.47)	1						
2. Gender		−0.18	1					
3. Trait modesty	27.64 (3.57)	−0.28	0.21	1				
4. Self‐esteem	28.85 (3.26)	0.05	0.47**	−0.19	1			
5. Expressive suppression	3.63 (1.31)	0.24	−0.37*	−0.32*	−0.24	1		
6. Cognitive reappraisal	5.25 (0.83)	0.11	0.05	−0.04	0.22	−0.03	1	
7. Fear of negative	3.25 (0.96)	0.18	−0.19	−0.17	−0.08	0.43**	0.10	1
8. Social interaction anxiety	2.79 (0.65)	0.18	−0.31*	−0.17	−0.30*	0.45**	−0.43**	0.38**

*Note:* *p < 0.05, **p < 0.01.

#### 3.1.2 Modesty and Social Feedback

Results showed that the participants' expectation of social feedback had an optimistic bias. The average percentage of predicted acceptance feedback before experiment was 0.69, significantly higher than the random level of 0.5 (*t* = 9.01, *p* < 0.001, *d* = 1.23). And the percentage of recalled acceptance feedback was 0.65, which was also significantly higher than the random level (*t* = 9.96, *p* < 0.001, *d* = 1.50). The correlation between trait modesty and these two variables was not significant (*p*s > 0.05). The average trial times and reaction times of each experimental condition are shown in Table 2. During the task, the percentage of expected acceptance feedback was significantly higher than that expected rejection feedback, *F*_(1,46)_ = 14.65, *p* < 0.001. Participants' response time to the acceptance feedback was significantly lower compared to the rejection feedback, *F*_(1,46)_ = 12.33, *p* = 0.001.

**TABLE 2:** The average trial number and reaction time of each experimental condition.

Conditions	Average trial number (SD)	Reaction time (s)
YES (participants' prediction)	90.81 (20.73)	1.12 (0.26)
YES–YES	45.34 (10.42)	1.12 (0.27)
YES–NO	45.47 (10.68)	1.11 (0.26)
NO (participants' prediction)	67.43 (21.20)	1.18 (0.25)
NO–YES	33.68 (10.71)	1.17 (0.27)
NO–NO	33.74 (10.88)	1.18 (0.24)

#### 3.1.3 Modesty and Emotional State

**FIGURE 2:**
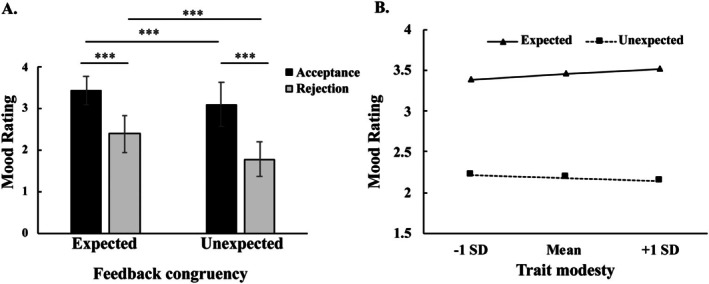
Mood rating and its relationship with trait modesty during the experiment. (A) showed participants’ mood rating under various conditions during the experiment, while (B) showed the moderation of trait modesty between feedback congruency and mood ratings. ***p < 0.001.

There was no statistically significant correlation between trait modesty and self‐esteem, positive affect, and negative affect before and after the experiment (*p*s > 0.05). As for mood rating, a 2 (feedback valence: acceptance, rejection) × 2 (feedback congruency: expected, unexpected) repeated measure ANOVA showed that the main effect of feedback valence was significant, *F*_(1,46)_ = 71.14, *p* < 0.001, η_p_^2^ = 0.61, indicating that the participants' mood of acceptance feedback (M = 2.91) was significantly higher than that of rejection feedback (M = 2.44). There was also a significant main effect of feedback congruency, *F*_(1,46)_ = 200.17, *p* < 0.001, η_p_^2^ = 0.81, indicating that the emotional score of expected condition (M = 3.26) was significantly higher than that of unexpected condition (M = 2.09). The interaction between feedback valence and feedback congruency was significant (see Figure 2A), *F*_(1,46)_ = 14.35, *p* < 0.001, η_p_^2^ = 0.24. Further simple effects analysis revealed that the emotional score of acceptation feedback was much higher than that of rejection feedback in the expected condition, *F*_(1,46)_ = 127.27, *p* < 0.001, η_p_^2^ = 0.74, and in the unexpected condition, *F*_(1,46)_ = 18.46, *p* < 0.001, η_p_^2^ = 0.29. Moreover, the mood rating of expected condition was higher than unexpected condition in rejection condition, *F*_(1,46)_ = 161.85, *p* < 0.001, η_p_^2^ = 0.78, and the acceptation condition. *F*_(1,46)_ = 185.38, *p* < 0.001, η_p_^2^ = 0.80.

Hierarchical linear modeling (HLM) analysis was conducted to investigate the moderating effect of trait modesty on feedback congruency, feedback valence, and their interaction. The Level‐1 model of HLM included feedback congruency and feedback valence, and the Level‐2 model included trait modesty scores (grand‐mean centered). Results showed that the significant effect of feedback congruency, β_1_ = 0.61, *t*_(46)_ = 11.40, *p* < 0.001, feedback valence, β_2_ = 1.31, *t*_(46)_ = 12.86, *p* < 0.001, and the interaction of them, β_3_ = −0.28, *t*_(46)_ = −3.83, *p* < 0.001. The results of model 2 showed that trait modesty had a significant interaction with feedback congruency, γ_11_ = 0.03, *t*_(45)_ = 2.41, *p* = 0.02, suggesting that trait modesty enhanced the positive predictive effect of congruency on mood rating (see Table 3). Simple slope test (Figure 2B) showed that expected feedback was rated more positively than unexpected feedback in the high modesty group (*b* = 1.38, *t* = 2.50, *p* = 0.020). Similarly, in the low modesty group, the mood rating was more positive in expected feedback than unexpected feedback (*b* = 1.17, *t* = 2.71, *p* = 0.010), but the trend of this difference was smaller than in the high modesty group.

**TABLE 3:** The results of Model 2 in HLM analysis of mood rating.

Variables	Beta	SE	*t*	df	*p*
Intercept (β_0j_)					
Trait modesty (γ_01_)	−0.02	0.02	−1.17	45	0.25
Feedback congruency (β_1j_)					
Trait modesty (γ_11_)	0.03	0.01	2.41	45	0.02*
Feedback valence (β_2j_)					
Trait modesty (γ_21_)	0.02	0.03	0.61	45	0.54
Interaction (β_3j_)					
Trait modesty (γ_31_)	−0.01	0.02	−0.41	45	0.68

*Note:* *p < 0.05.

### 3.2 Image Analysis

#### 3.2.1 Feedback Congruency

There was a significant effect of feedback congruency (expected > unexpected) on brain activation in several regions, including putamen, vmPFC, precentral gyrus (preCG), postcentral gyrus, middle cingulate cortex (MCC), supplementary motor area (SMA), dorsolateral prefrontal cortex (DLPFC), ventral lateral prefrontal cortex (VLPFC), IPL, paracentral gyrus, superior temporal gyrus (STG), middle temporal gyrus (MTG), and precuneus. On the contrary, there was a significant effect of feedback congruency (unexpected > expected) on brain activation in several regions, including MTG, SMA, dorsomedial prefrontal cortex (DMPFC), anterior insular cortex, dACC, preCG, middle frontal gyrus (MFG), and superior frontal gyrus (SFG).

#### 3.2.2 Feedback Valence

There was a significant effect of feedback valence (acceptance > rejection) on brain activation in several regions involved in social–emotional and reward processing, including vmPFC, vACC, caudate nucleus, medial orbital frontal cortex (medial OFC), and posterior insular cortex (PIC). Moreover, acceptance versus rejection also activated the social cognition network involved in self‐reflective processing and social reasoning and mentalization processing, such as bilateral DMPFC, temporo‐parietal junction (TPJ), posterior cingulate cortex, STG, MTG, IPL, and precuneus. On the contrary, there was a significant effect of feedback valence (rejection > acceptance) on brain activation in several regions, including postcentral gyrus, SFG, SPL, SMA, MCC, and PIC.

#### 3.2.3 Interaction

Contrasting rejection to acceptance (unexpected > expected) yielded increased activation in calcarine, cuneus, and MFG. Moreover, contrasting rejection to acceptance (unexpected > expected) yielded increased activation in several regions, including bilateral TPJ, bilateral IPL, temporal pole, STG, MTG, bilateral SMA, bilateral DMPFC, bilateral DLPFC, and bilateral VLPFC.

#### 3.2.4 The Moderation of Trait Modesty

**FIGURE 3:**
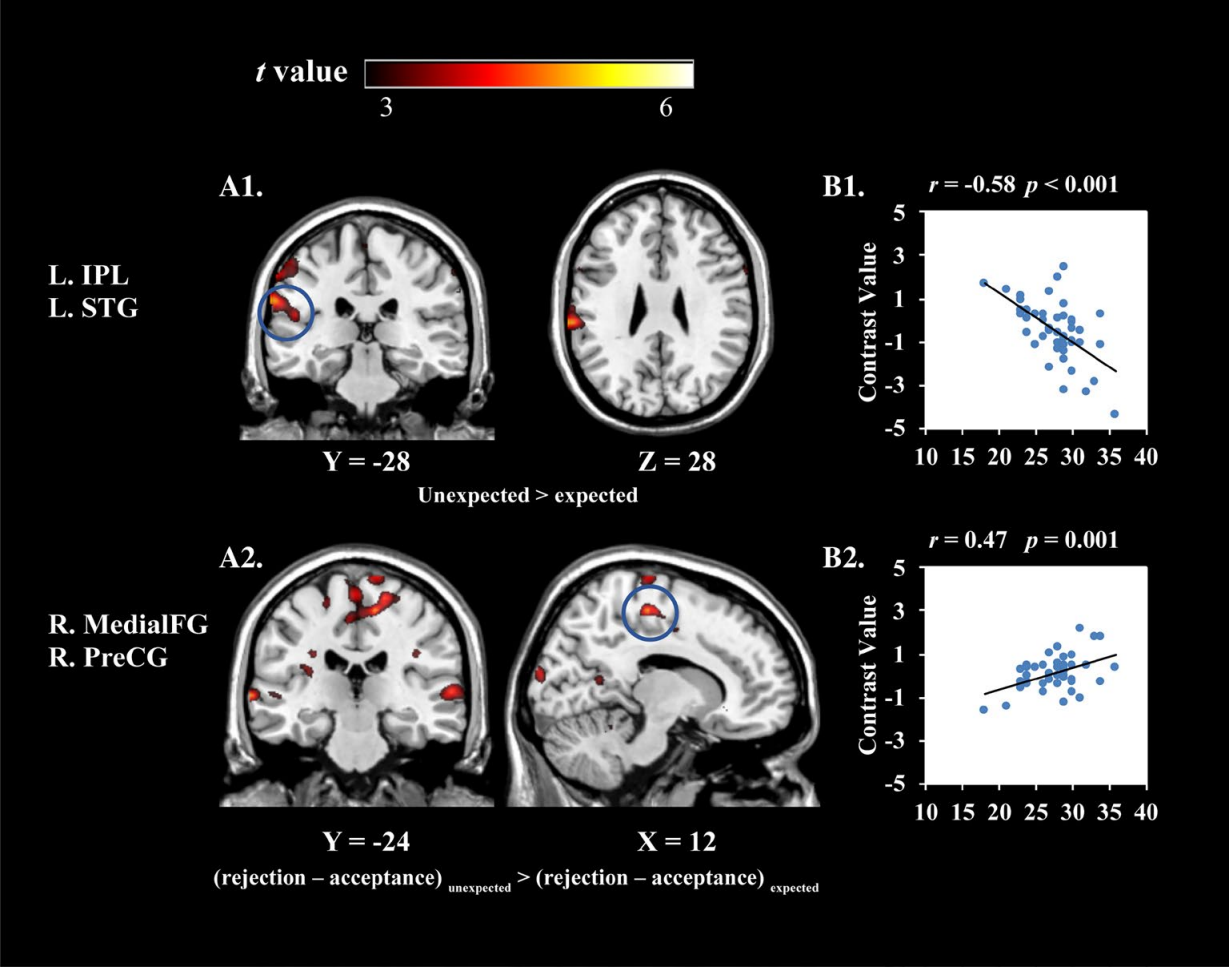
Interaction of trait modesty and feedback congruency in the whole‐brain MLM analysis. (A1) Brain activation map of negative correlation between trait modesty and unexpected versus expected feedback in left IPL and STG; (B1) Correlation between IPL brain region pair ratio and trait modesty; (A2) Brain activation map of positive correlation between trait modesty and feedback congruency [(rejection—acceptance) _unexpected_ > (rejection—acceptance) _expected_] in right MedialFG and preCG; (B2) Correlation between MedialFG brain region pair ratio and trait modesty. L: Left; R: Right. Render the image using the CH2 template in MRIcron.

To investigate how trait modesty moderated the feedback congruency, feedback valence, and their interaction, we included the trait modesty score as a covariable in the group analysis and conducted a whole‐brain regression analysis, excluding the effects of age, gender, and self‐esteem. Therefore, there were (see Figure 3 and Table A of Supporting Information): (1) a negative correlation between trait modesty and feedback congruency (unexpected > expected) on brain activation in IPL and STG; (2) a positive correlation between trait modesty and feedback valence (acceptance > rejection) on brain activation in several regions, including bilateral STG, TPJ, bilateral MTG, angular, medial prefrontal cortex (mPFC), bilateral DMPFC, dACC, vmPFC/vACC, and DLPFC; (3) and a positive correlation between trait modesty and the interaction of feedback valence and feedback congruency [(rejection–acceptance)_unexpected_ > (rejection–acceptance)_expected_] on brain activation in several regions, which included medial frontal gyrus (MedialFG), preCG, SMA, and paracentral gyrus.

### 3.3 PPI Analysis

**FIGURE 4:**
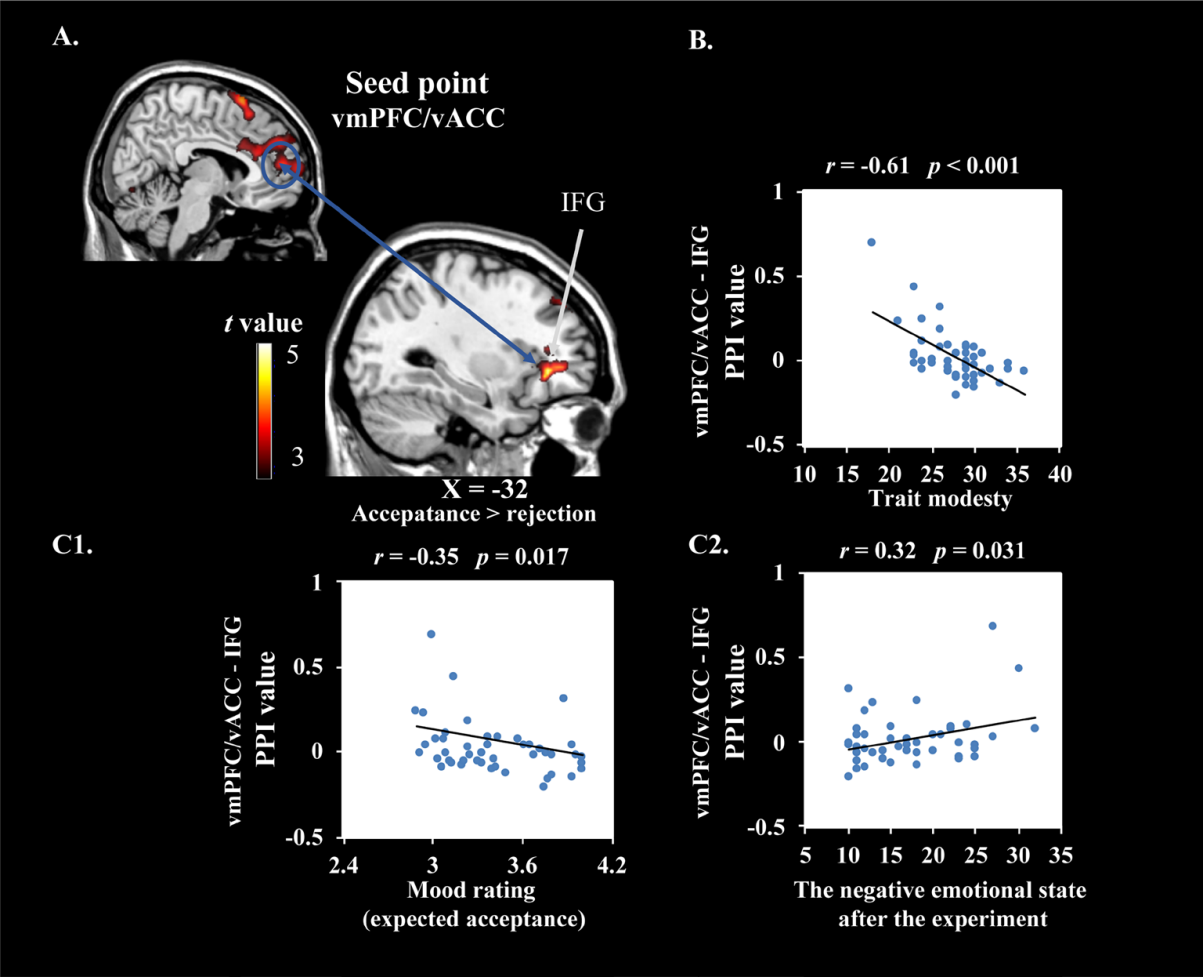
Results of PPI whole‐brain analysis of vmPFC/vACC seed point. (A) Regions negatively correlated to vmPFC/vACC under the moderation of feedback valence (acceptance > rejection); (B) The negative correlation of PPI values of vmPFC/vACC‐IFG between feedback valence (rejection > acceptance) with trait modesty; (C) The correlation of the PPI value of vmPFC/vACC‐IFG with mood rating under the expected acceptance condition (C1) and the negative emotional state after the experiment (C2).

Results showed a negative PPI in the right vmPFC/vACC and left inferior frontal gyrus (IFG) moderated with the feedback valence (acceptance > rejection). Negative PPI results indicated that neural activity in the vmPFC/vACC was enhanced while neural activity in the left IFG was weakened (see Table 4), and this negative relationship was stronger in the acceptance condition (compared to the rejection condition). Trait modesty was significantly negatively correlated with the PPI values between the vmPFC/vACC and IFG. To reveal whether the functional connectivity of vmPFC/vACC‐IFG was negative or positive in different modest groups, the values of the peak coordinates of the brain regions of the IFG were extracted and compared with 0. Results showed that the functional connectivity of vmPFC/vACC‐IFG was significantly lower than 0 in the high modesty group (*t* = −2.68, *p* = 0.014, *d* = 0.54) and higher than 0 in the low modesty group (*t* = 2.52, *p* = 0.021, *d* = 0.57). In addition, the correlation between the PPI value and the mood rating was calculated (see Figure 4C), and the correlation analysis between the functional connectivity strength of vmPFC/vACC‐IFG and the mood rating showed that the PPI value of vmPFC/vACC‐IFG was significantly negatively correlated to the mood rating under the expected acceptance condition (*r* = −0.37, *p* = 0.013) and positively correlated to the negative emotional state after the experiment (*r* = 0.32, *p* = 0.031).

**TABLE 4:** Results of PPI whole‐brain analysis of vmPFC/vACC seed point, all with *p* < 0.05 (FEW‐corrected at the cluster level), with side, BA, MNI coordinates, *t*‐values Z and cluster size (k). Only significant effects are listed. For each cluster, the maximum peak in gray matter is reported.

Region	Side	BA	MNI			*t*	Z	k
			x	y	z			
Trait modesty was negatively correlated with PPI								
Frontal_Inf_Orb/IFG	L		−32	34	−4	4.79	4.28	505
Sub‐Gyral	L		−18	26	22	4.5	4.06	
MFG	L		−32	40	−2	4.24	3.86	
ACC	L		−14	30	22	2.73	—	

### 3.4 Brain Behavior Correlation

**FIGURE 5:**
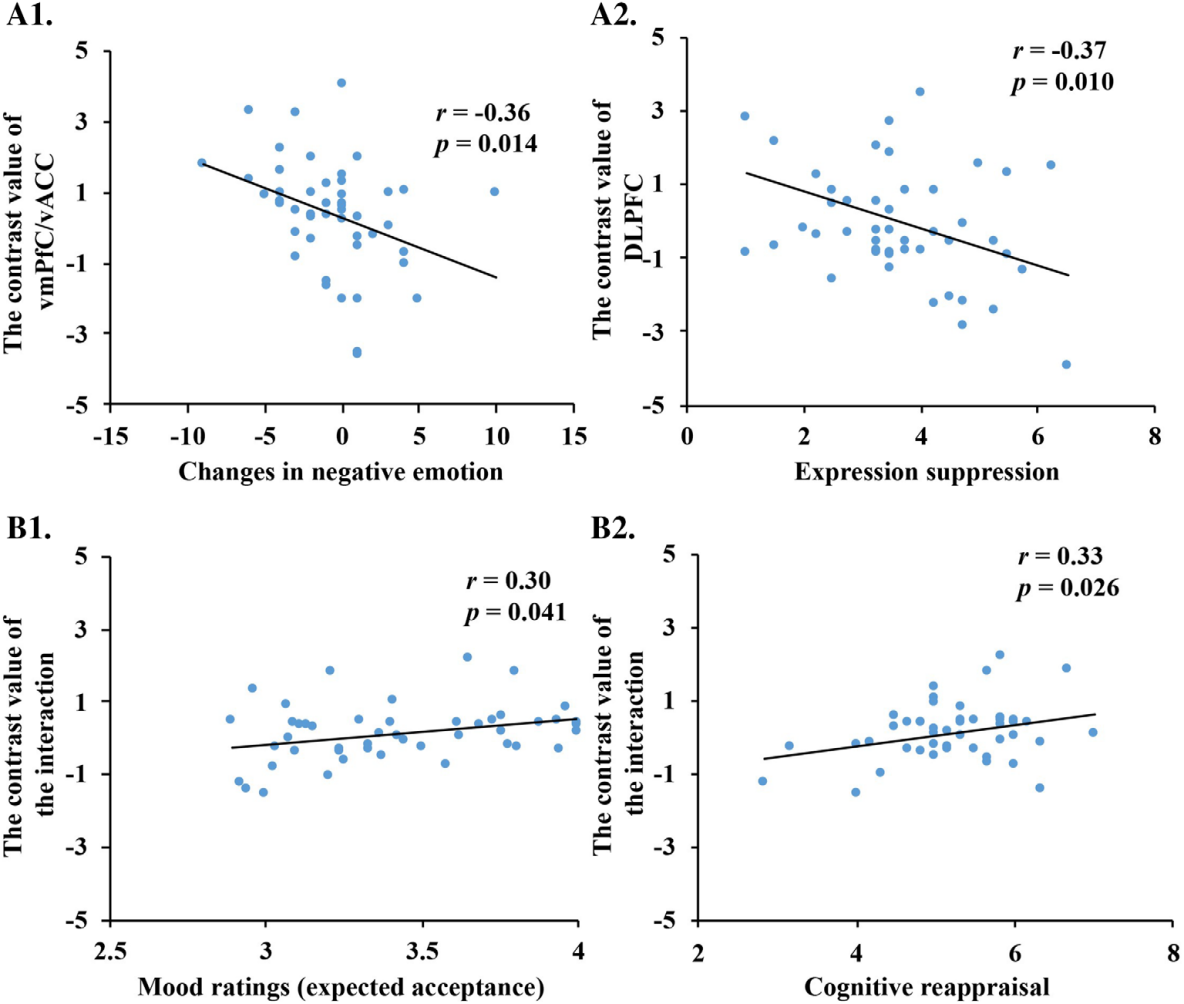
Results of the correlation analysis between brain neural activity and behavior. (A1 and A2) the correlation analysis of the difference in neural activity intensity in the right vmPFC/vACC and the left DLPFC between the feedback valence (acceptance > rejection) and changes in negative emotions and expression suppression; (B1 and B2) the correlation analysis results of the difference in neural activity intensity in the right MedialFG due to the interaction between feedback valence and congruency, with the differences in mood ratings under the expected acceptance condition and cognitive reappraisal.

We extracted the contrast values of significantly activated brain regions under various conditions, and performed correlation analysis between these values and emotion regulation strategies, changes in emotional states before and after the experiment, and mood ratings (see Figure 5). Results showed that the difference in neural activity intensity in the right vmPFC/vACC between the feedback valence (acceptance > rejection) was significantly negatively correlated with changes in negative emotion. That is to say, the stronger the neural activity intensity in the vmPFC/vACC under the acceptance feedback compared to the rejection feedback, the less negative emotion the participants felt after the experiment. The difference in neural activity intensity in the left DLPFC between the feedback valence (acceptance > rejection) was significantly negatively correlated with individual differences in expression suppression. The interaction between feedback valence and feedback congruency showed that the difference in neural activity intensity in the right MedialFG was significantly positively correlated with individual differences in cognitive reappraisal. These results suggested that individuals with less expression suppression exhibited stronger neural activity intensity in the DLPFC under the acceptance feedback compared to the rejection feedback. Individuals with more cognitive reappraisal showed stronger neural activity intensity in the MedialFG under unexpected rejection feedback compared to the unexpected acceptance feedback. Additionally, mood ratings under the expected acceptance feedback were significantly positively correlated with MedialFG.

## 4 Discussion

In this study, we explored the cognitive and emotional responses and neural mechanisms of modest individuals during the processing of different social feedback. Behaviorally, modest individuals experienced more positive emotions in response to expected feedback. Self‐reported questionnaires also indicated that trait modesty was negatively correlated with the emotion regulation strategy of expressive suppression, suggesting that the reduced negative emotions in modest individuals were not due to the inhibition of emotional expression. At the neural level, results suggested that trait modesty was associated with more adaptive cognitive‐emotional processing of social feedback. Results showed that the more modest the participant was, the weaker the activation in the IPL and left STG under unexpected conditions compared to expected conditions. Additionally, under acceptance versus rejection conditions, trait modesty was positively correlated with activation in regions including the vmPFC/vACC, dACC, and DLPFC, which were involved in cognitive processes such as social rewards, self‐information, and social information processing. PPI and brain‐behavior correlation analyses of the vmPFC/vACC and MedialFG further revealed the mechanisms of modesty helping individuals reduce negative experiences and enhance positive experiences. These findings indicated that modest individuals had more positive experiences and fewer negative experiences when receiving different social feedback. The results contribute to further understanding the advantages of modesty in social information processing and help improve people's responses to social feedback.

### 4.1 Modesty Helps Face Rejected or Unexpected Feedback More Calmly

The study indicates that modesty helps individuals respond more calmly to the rejected or unexpected social feedback, and this effect is not operated through expressive suppression. The fMRI results indicated that modesty modulated brain activity when individuals received unexpected feedback. Across all participants, unexpected feedback, compared to expected feedback, significantly activated brain regions such as the dACC, DMPFC, and MTG. Importantly, trait modesty was negatively correlated with the main effect of feedback congruency (unexpected vs. expected) in brain regions such as the IPL and left STG. Previous research has suggested that the IPL is associated with self‐relevant information, such as personality trait evaluations (Kircher et al. 2000) and attentional processing (Culham and Kanwisher 2001). In addition, it has been identified as a core region of the mirror neuron system, playing a critical role in distinguishing information of self and others (Fogassi et al. 2005). For instance, Kircher et al. (2000) reported stronger IPL activation when participants processed information about themselves compared to information about their partners. As for the STG, it is mainly involved in the integration and processing of social information and shows stronger activation during social information processing (e.g., Lee Masson et al. 2024; Wilson‐Mendenhall et al. 2013; Zahn et al. 2007). Taken together with the self‐report results (trait modesty was negatively correlated with expressive suppression), these results suggest that when receiving social feedback inconsistent with their expectations, modest individuals pay less attention to self‐relevant social information rather than suppressing the expression of their negative emotions. These findings are also consistent with theoretical perspectives on modesty, which conceptualize modesty as a trait characterized by low self‐focus (Nadelhoffer and Wright 2017; Peterson and Seligman 2004).

Besides, PPI and brain‐behavior correlation analyses of the vmPFC/vACC further revealed the mechanisms of modesty helping individuals reduce negative experiences. Under acceptance conditions compared to rejection conditions, the more modest the participant was, the stronger the activation in the vmPFC/vACC. Brain‐behavior correlation analyses indicated a significant negative correlation between individual differences in vmPFC/vACC neural activity across conditions and levels of negative emotional state (after‐experiment minus before‐experiment). Previous research indicates that vmPFC activation can promote positive reappraisal of negative emotional events and enhance positive emotional experiences (Doré et al. 2017), so these results suggested that social acceptance holds high subjective reward value for modest individuals, which can reduce negative emotional experiences. Moreover, the PPI analysis, using the vmPFC/vACC as a seed point, revealed the negative connectivity between vmPFC/vACC and left IFG in the high‐modesty group (vs. positive connectivity in the low‐modesty group). Further analyses demonstrated that the functional connectivity between vmPFC/vACC and IFG was negatively associated with emotion ratings under expected acceptance conditions, and positively associated with post‐experiment negative emotional experiences. The IFG plays an inhibitory role in cognitive and emotional processing (Berkman et al. 2009), with emotion suppression linked to increased IFG neural activity (Goldin et al. 2008). This vmPFC/vACC–IFG connectivity may reflect a “top‐down” cognitive emotional regulation mechanism, whereby the value appraisal system (vmPFC/vACC) modulates the inhibitory control region (IFG). For example, a previous study found that stronger positive vmPFC–IFG functional connectivity was significantly correlated with a higher proportion of lying behavior, as individuals inhibited their honesty for greater rewards (Yin and Weber 2019). Therefore, the PPI results suggest that when facing negative social feedback, modest individuals are less likely to rely on expressive suppression and instead engage in positive reappraisal to regulate their emotions. These findings align with previous research suggesting that modest individuals tend to adopt a more open attitude and effectively manage negative information (Chancellor and Lyubomirsky 2013; Exline et al. 2008).

### 4.2 Modesty Enhances Positive Experiences Derived From Accepted or Expected Feedback

In addition to reducing negative experiences, modesty may also enhance positive experiences derived from favorable social feedback. On the one hand, modesty enhanced individuals’ positive experiences in response to expected feedback. Behaviorally, all individuals exhibited an optimism bias in their expectations of social acceptance feedback, predicting that they would receive more acceptance feedback than random levels. Moreover, HLM analysis indicated that modesty enhanced individuals’ positive emotional experiences in response to expected feedback. Besides, modesty was also positively correlated with neural activity in the MedialFG and preCG brain regions in response to the interaction between expectation consistency and feedback valence [(rejection > acceptance)_unexpected_ > (rejection > acceptance)_expected_]. And brain–behavior correlation analyses revealed that neural activity in the MedialFG positively correlated with emotional ratings under expected acceptance conditions. Previous studies have shown that brain regions such as the MedialFG and preCG are involved in self‐reflection and cognitive emotion regulation (Herwig et al. 2010; Kohn et al. 2014; McLellan et al. 2012). Thus, these results suggest that modest individuals value others’ approval and feel stronger positive emotions when receiving expected social feedback, especially expected acceptance.

On the other hand, modesty enhances individuals’ positive experiences in response to acceptance feedback. For all participants, acceptance feedback, compared to rejection feedback, significantly activated regions such as the vmPFC/vACC, medial OFC, caudate nucleus, putamen, TPJ, and precuneus. The vmPFC and vACC were involved in reward and emotional processing related to social evaluation, reflecting the integration of positive social emotions or the subjective value of social rewards. In contrast, rejection feedback, compared to acceptance feedback, significantly activated brain regions such as the SPL, SMA, MCC, and PIC, which are involved in the processing of social pain (Eisenberger et al. 2003; Zeidan et al. 2015). Moreover, modesty was positively correlated with the main effect of feedback valence (acceptance vs. rejection) in some brain regions associated with social cognitive processing and emotional regulation, such as STG, MTG, angular, mPFC, and DMPFC. Previous research has indicated that the social cognitive brain network, composed of regions such as the STG, MTG, mPFC, and DMPFC, plays a crucial role in the processing of social information and is closely related to the abilities of mentalizing and social reasoning, including perceiving and understanding others states, intentions, emotions, and behaviors (Donaldson et al. 2015; Mitchell et al. 2005; Powers et al. 2013; Santiesteban et al. 2012; Tholen et al. 2020; van Schie et al. 2018, 2020; Zahn et al. 2007). Moreover, previous studies on modesty have shown that modest individuals pay more attention to the states of others and are willing to establish connections and maintain harmonious interpersonal relationships (Nadelhoffer and Wright 2017; Worthington et al. 2021). Our findings provide further neural evidence suggesting that modest individuals possess a stronger motivation for social interaction and experience stronger positive emotions when receiving acceptance feedback.

### 4.3 Approaching Modesty to Improve Social Feedback Responses

From the perspective of social feedback, our study offers insights into how individuals can better process social feedback information. Social feedback is crucial to individuals’ self‐concept (Lundgren 2004; Peters et al. 2024). Due to cognitive biases in self‐perception (Alicke and Sedikides 2009; Korn et al. 2012), individuals often experience negative emotions when receiving negative or unexpected feedback, prompting them to adopt suppressive strategies to reduce such experiences (Gross 2002, 2015), although these strategies may also diminish positive emotional experiences (Fernandes and Tone 2021). By overcoming these biases, modest individuals demonstrate advantages in processing social feedback and maintaining higher levels of psychological well‐being (Worthington et al. 2021; Zheng and Wu 2020).

Therefore, people can approach modesty from two perspectives to improve their responses to social feedback. First, people can learn from the cognitive processing strategies of modest individuals when facing social feedback, especially in dealing with unexpected rejection. By reducing self‐focus, individuals can minimize negative experiences. Second, modesty is not only a trait but can also function as a temporary state (Chancellor and Lyubomirsky 2013; Wang et al. 2025). Individuals can temporarily increase their level of modesty by recalling their modest experiences (Kesebir 2014; Wang et al. 2025). Thus, when receiving social feedback, temporarily priming one's modesty state can help in better coping with the feedback.

### 4.4 Limitations and Future Directions

However, there are some limitations in our study. First, participants in this study were young Chinese adults, which may limit the generalizability of the findings. Previous research indicates that sample characteristics can influence how social feedback affects individual emotions. For example, some studies have shown that Chinese participants are more strongly affected by negative social feedback (Hu et al. 2015; Rapp et al. 2021). Future research should further explore the potential effects of sample characteristics including culture, age, and gender. Second, this study utilized the SJP, where individuals received single evaluations from multiple others. In real social interactions, however, interactions with others, especially significant others, are often reciprocal. Future research could design social feedback scenarios involving multiple rounds of interaction to further examine the cognitive processing patterns and neural activities of modest individuals in such contexts. Third, the impact of social feedback on individuals is long‐term (Dobbelaar et al. 2023), and the effects of some emotion regulation strategies also manifest over the long course of life (Brans et al. 2013). Future studies could investigate the moderating role of trait modesty in the long‐term effects of social interaction information, particularly concerning the adaptability of adolescents to social feedback. Finally, considering the characteristics of modesty (Nadelhoffer and Wright 2017; Peterson and Seligman 2004), we posit that its positive effects may extend beyond social feedback contexts. Future research could test this proposition in diverse scenarios—such as individuals' responses to personal success or failure—to further validate the value of modesty.

## 5 Conclusions

In conclusion, this study examined the patterns and neural mechanisms of modest individuals when processing social feedback information. Results showed that modest individuals were less likely to use the expressive suppression strategy and that the trait modesty modulated brain activity in regions associated with self‐relevant information processing and cognitive emotion regulation. Trait modesty modulated brain activity in the IPL and left STG under unexpected conditions compared to expected conditions, as well as in the vmPFC/vACC, dACC, and DLPFC under acceptance versus rejection conditions. Additionally, PPI and brain‐behavior correlation analyses of the vmPFC/vACC and MedialFG further revealed the mechanisms of modesty helping individuals reduce negative experiences and enhance positive experiences.

## Ethics Statement

This study was approved by the Committee for Protecting Human and Animal Subjects of the School of Psychological and Cognitive Sciences, Peking University.

## Consent

All participants provided written informed consent prior to participating in the study.

## Supporting information


**TABLE A:** Differences in whole‐brain activation related to trait modesty, all with *p* < 0.05 (FWE‐corrected at the cluster level), with BA, side, MNI coordinates, *t*‐values Z and cluster size. Only significant effects are listed. For each cluster, the maximum peak in gray matter is reported. Analyses controlled for age, gender, and self‐esteem.
**TABLE B:** Differences in whole‐brain activation related to trait modesty, all with *p* < 0.05 (FWE‐corrected at the cluster level), with BA, side, MNI coordinates, *t*‐values Z and cluster size. Only significant effects are listed. For each cluster, the maximum peak in gray matter is reported. Analyses controlled for age, gender, self‐esteem and social interaction anxiety.

## Data Availability

The data that support the findings of this study are available from the corresponding author upon reasonable request.

## Endnotes

^1^
Considering the potential influence of social interaction anxiety, we conducted additional analyses including it in the model. The results were broadly consistent with those without this covariate, and the main brain regions of interest still showed significant activation (see Table B of Supplementary data). To ensure clarity and maintain consistency with other analyses (e.g., PPI), the Results section reports findings from the analyses without social interaction anxiety included.
