# Supplementary material for: “Take the Rough With the Smooth”: Modesty Modulates Neurocognitive and Emotional Processing of Social Feedback
Source: Hum Brain Mapp. 2025 Oct 29;46(16):e70395. doi: 10.1002/hbm.70395 (PMC12570044; doi:10.1002/hbm.70395)
Supplement: Supplementary file 1 — TABLE A: Differences in whole‐brain activation related to trait modesty, all with p < 0.05 (FWE‐corrected at the cluster level), with BA, side, MNI coordinates, t‐values Z and cluster size. Only significant effects are listed. For each cluster, the maximum peak in gray matter is reported. Analyses controlled for age, gender, and self‐esteem. TABLE B: Differences in whole‐brain activation related to trait modesty, all with p < 0.05 (FWE‐corrected at the cluster level), with BA, side, MNI coordinates, t‐values Z and cluster size. Only significant effects are listed. For each cluster, the maximum peak in gray matter is reported. Analyses controlled for age, gender, self‐esteem and social interaction anxiety. [file HBM-46-e70395-s001.docx]

**Table A**. Differences in whole-brain activation related to trait modesty, all with *p* < 0.05 (FWE-corrected at the cluster level), with BA, side, MNI coordinates, T-values Z and cluster size. Only significant effects are listed. For each cluster, the maximum peak in gray matter is reported. Analyses controlled for age, gender, and self-esteem.

|  |  |  | **MNI** | | |  |  |  |
| --- | --- | --- | --- | --- | --- | --- | --- | --- |
| **region** | **side** | **BA** | ***x*** | ***y*** | ***z*** | ***T*** | ***Z*** | **Cluster** |
| **negative correlation between trait modesty and feedback congruency (**unexpected > expected**)** | | | | | | | | |
| IPL | L |  | -68 | -28 | 28 | 4.86 | 4.31 | 206 |
| STG | L | 40 | -50 | -28 | 18 | 3.97 | 3.63 |  |
| **positive correlation between trait modesty and feedback congruency (**unexpected > expected**)** | | | | | | | | |
| None |  |  |  |  |  |  |  |  |
| **positive correlation** **between trait modesty and feedback valence (acceptance > rejection)** | | | | | | | | |
| STG | R |  | 58 | -20 | -4 | 6.70 | 5.50 | 2600 |
| Cerebellum_Crus2 | R |  | 30 | -78 | -40 | 4.94 | 4.36 |  |
| TPJ | R | 40 | 58 | -48 | 26 | 4.59 | 4.11 |  |
| MTG | R | 21/22 | 68 | -28 | -6 | 4.55 | 4.08 |  |
| ITG | R |  | 66 | -48 | 18 | 4.20 | 3.82 |  |
| Angular gyrus | R |  | 60 | -56 | 30 | 4.17 | 3.79 |  |
| SMA | R |  | 8 | 18 | 66 | 5.17 | 4.52 | 471 |
| SFG | R |  | 8 | 24 | 54 | 4.83 | 4.28 |  |
| mPFC | L |  | 0 | 26 | 60 | 4.81 | 4.27 |  |
| DMPFC | L | 9 | -8 | 52 | 28 | 4.83 | 4.28 | 765 |
| DMPFC | R |  | 4 | 50 | 32 | 4.28 | 3.88 |  |
| dACC | R | 32/24 | 6 | 32 | 22 | 4.14 | 3.77 |  |
| Cerebellum_Crus2 | L |  | -40 | -64 | -42 | 4.67 | 4.16 | 458 |
| SFG | L |  | -28 | 60 | 28 | 4.39 | 3.96 | 405 |
| DLPFC | L | 46/9 | -48 | 42 | 24 | 4.31 | 3.90 |  |
| MFG | L |  | -42 | 40 | 30 | 3.85 | 3.54 |  |
| vmPFC/vACC | R | 10/32 | 6 | 54 | 12 | 4.30 | 3.89 | 227 |
| MTG | L | 21 | -64 | -32 | 2 | 4.29 | 3.88 | 239 |
| STG | L |  | -56 | -38 | 10 | 3.75 | 3.46 |  |
| **negative correlation between trait modesty and feedback valence (acceptance > rejection)** | | | | | | | | |
| None |  |  |  |  |  |  |  |  |
| **positive correlation between trait modesty and interaction of feedback valence and feedback congruency [(rejection > acceptance) _unexpected_ > (rejection > acceptance) _expected_]** | | | | | | | | |
| MedialFG | R |  | 12 | -24 | 58 | 4.82 | 4.28 | 360 |
| Precentral gyrus | R |  | 18 | -22 | 66 | 4.21 | 3.82 |  |
| SMA | R | 6 | 2 | -10 | 52 | 4.17 | 3.79 |  |
| Paracentral lobule | L | 6 | -6 | -30 | 54 | 3.89 | 3.57 |  |
| **negative correlation between trait modesty and interaction of feedback valence and feedback congruency [(rejection > acceptance) _unexpected_ > (rejection > acceptance) _expected_]** | | | | | | | | |
| None |  |  |  |  |  |  |  |  |

Notes: IPL = inferior parietal lobe; STG = superior temporal gyrus; TPJ = temporo-parietal junction; MTG = middle frontal gyrus; ITG = inferior temporal gyrus; SMA = supplementary motor area; SFG = superior frontal gyrus; mPFG = medial prefrontal cortex; DMPFC = dorsomedial prefrontal cortex; dACC = dorsal anterior cingulate cortex; DLPFC = dorsolateral prefrontal cortex; MFG = middle frontal gyrus; vmPFC = ventral medial prefrontal cortex; MedialFG = medial frontal gyrus.

**Table B.** Differences in whole-brain activation related to trait modesty, all with *p* < 0.05 (FWE-corrected at the cluster level), with BA, side, MNI coordinates, T-values Z and cluster size. Only significant effects are listed. For each cluster, the maximum peak in gray matter is reported. Analyses controlled for age, gender, self-esteem and social interaction anxiety.

|  |  |  | **MNI** | | |  |  |  |
| --- | --- | --- | --- | --- | --- | --- | --- | --- |
| **region** | **side** | **BA** | ***x*** | ***y*** | ***z*** | ***T*** | ***Z*** | **Cluster** |
| **negative correlation between trait modesty and feedback congruency (**unexpected > expected**)** | | | | | | | | |
| IPL | L |  | -66 | -28 | 28 | 4.69 | 4.17 | 79 |
| Postcentral Gyrus | L |  | -66 | -18 | 32 | 3.56 | 3.30 |  |
| **positive correlation between trait modesty and feedback congruency (**unexpected > expected**)** | | | | | | | | |
| None |  |  |  |  |  |  |  |  |
| **positive correlation between trait modesty and feedback valence (acceptance > rejection)** | | | | | | | | |
| STG | R |  | 58 | -20 | -4 | 6.18 | 5.16 | 2779 |
| Cerebellum_Crus1 | R |  | 30 | -78 | -32 | 4.98 | 4.38 |  |
| TPJ | R | 40 | 68 | -48 | 4 | 4.96 | 4.36 |  |
| SMA | R |  | 10 | 18 | 66 | 5.22 | 4.54 | 1553 |
| SFG | L |  | -8 | 52 | 30 | 5.10 | 4.46 |  |
| Cerebellum_Crus1 | L |  | -40 | -70 | -34 | 4.89 | 4.31 | 1085 |
| STG | L |  | -50 | -28 | -2 | 4.85 | 4.29 | 386 |
| MTG | L |  | -64 | -54 | 0 | 4.24 | 3.84 |  |
| MTG | L |  | -64 | -32 | 4 | 4.21 | 3.82 |  |
| STG | L |  | -46 | 6 | -18 | 4.76 | 4.22 | 259 |
| STG | L | 38 | -52 | 20 | -14 | 4.55 | 4.07 |  |
| STG | L |  | -46 | 24 | -18 | 4.37 | 3.94 |  |
| STG | R |  | 44 | -40 | 4 | 4.75 | 4.22 | 92 |
| Precentral Gyrus | L |  | -36 | -12 | 40 | 4.73 | 4.2 | 81 |
| MedialFG |  |  | -12 | -14 | 60 | 4.72 | 4.19 | 223 |
| SFG |  |  | -20 | -8 | 74 | 4.10 | 3.73 |  |
| MFG |  |  | -30 | 2 | 68 | 3.65 | 3.38 |  |
| IFG |  |  | 52 | 30 | 18 | 4.68 | 4.16 | 159 |
| IPL | R |  | 56 | -50 | 42 | 4.40 | 3.96 | 196 |
| Parietal Lobe | L | 7 | -20 | -52 | 56 | 4.37 | 3.93 | 80 |
| MFG | L | 46 | -50 | 40 | 22 | 4.28 | 3.87 | 121 |
| SFG |  |  | -28 | 58 | 30 | 4.27 | 3.86 | 250 |
| MFG |  |  | -30 | 44 | 28 | 4.21 | 3.81 |  |
| Precuneus | L |  | -8 | -60 | 70 | 4.13 | 3.76 | 157 |
| Postcentral Gyrus | L | 5 | -6 | -48 | 66 | 3.73 | 3.44 |  |
| Postcentral Gyrus | L |  | -14 | -48 | 72 | 3.34 | 3.12 |  |
| vmPFC/vACC | R |  | 6 | 54 | 14 | 4.08 | 3.71 | 159 |
| MedialFG | R | 10 | 6 | 64 | 8 | 3.87 | 3.55 |  |
| STG | R | 38 | 50 | 20 | -14 | 3.95 | 3.61 | 110 |
| **negative correlation between trait modesty and feedback valence (acceptance > rejection)** | | | | | | | | |
| None |  |  |  |  |  |  |  |  |
| **positive correlation between trait modesty and interaction of feedback valence and feedback congruency [(rejection > acceptance) _unexpected_ > (rejection > acceptance) _expected_]** | | | | | | | | |
| MedialFG | R |  | 10 | -26 | 58 | 5.01 | 4.40 | 287 |
| Precentral gyrus | R |  | 18 | -22 | 64 | 4.10 | 3.73 |  |
| SMA | R | 6 | 2 | -20 | 56 | 4.01 | 3.66 |  |
| Paracentral Lobule | R | 6 | 4 | -36 | 70 | 3.89 | 3.57 | 120 |
| Cerebellum_4_5 | L |  | -20 | -44 | -20 | 4.41 | 3.96 | 141 |
| **negative correlation between trait modesty and interaction of feedback valence and feedback congruency [(rejection > acceptance) _unexpected_ > (rejection > acceptance) _expected_]** | | | | | | | | |
| None |  |  |  |  |  |  |  |  |

Notes: IPL = inferior parietal lobe; TPJ = temporo-parietal junction; IFG = Inferior Frontal Gyrus; STG = superior temporal gyrus; SFG = superior frontal gyrus; MTG = middle temporal gyrus; MedialFG = medial frontal gyrus; MFG = middle frontal gyrus; SMA = supplementary motor area.
